# Data on microbial diversity of camel milk microbiota determined by 16S rRNA gene sequencing

**DOI:** 10.1016/j.dib.2022.108744

**Published:** 2022-11-11

**Authors:** Rita Rahmeh, Abrar Akbar, Husam Alomirah, Mohamed Kishk, Abdulaziz Al-Ateeqi, Salah Al-Milhm, Anisha Shajan, Batool Akbar, Shafeah Al-Merri, Mohammad Alotaibi, Alfonso Esposito

**Affiliations:** a Environment and Life Sciences Research Centre, Kuwait Institute for Scientific Research, Kuwait; b International Centre for Genetic Engineering and Biotechnology, Trieste, Italy

**Keywords:** Camel milk, Metagenomics, Microbial diversity, Season, Geographical locations

## Abstract

Raw camel milk samples were collected from three geographical locations (south, north and middle Kuwait) during two seasons. Next generation sequencing of the V3-V4 regions of the 16S rRNA gene was used to analyze the bacterial community in camel milk. DNA was extracted from one hundred thirty-three samples, and libraries were prepared using custom fusion primers of the 16S rRNA gene and sequenced on Illumina HiSeq 2500 platform. 16S rRNA gene sequences were aligned against the SILVA database SSU release 138. The high-throughput sequencing data are available at the NCBI database under the Bioproject PRJNA814013. This work describes camel milk's bacterial diversity among different geographical locations and seasons. The distribution of alpha diversity measures among camel milk sample groups collected from different geographical locations and seasons is presented. A significant effect of these parameters on camel milk's bacterial diversity was shown. Linear discriminant analysis (LefSe) showed significant differentially abundant bacteria at the phylum, class, order, family and genus level among the three locations and seasons. LefSe identified a total of 83 and 40 differentially abundant genera in the different geographical locations and seasons, respectively. More details about the bacterial composition of raw camel milk at the phylum and genus level can be found in research article [1]. These data can be used to compare the diversity of milk bacterial community between different milk producing species and camels from different parts of the world. Besides, these findings will contribute to our understanding of the camel microbiome structure and might be useful for designing an appropriate control program in the camel dairy herd. The data described in this article are available in Mendeley Data [2].

**Specifications Table**

**Table:** 

Subject	Biology
Specific subject area	Metagenomics
Type of data	DNA sequences, Tables, figures
How the data were acquired	16S rRNA gene amplicon sequencing using Illumina HiSeq 2500 platform
Data format	Raw data, filtered data and analysed reads
Description of data collection	Samples of raw camel milk were collected from south, north and middle Kuwait during two seasons. The udder and the teats were disinfected by physical scrubbing with 70% ethylic alcohol and the first drops of camel milk were discarded. The samples were collected into sterile tubes and transported immediately to the laboratory for metagenomics DNA extraction. DNA was isolated using GenElute Bacterial Genomic DNA Kit. Libraries were prepared using custom fusion primers of the 16S rRNA gene and sequenced on Illumina HiSeq 2500 platform in 2 × 300 bp mode. Bioinformatics processing of the raw reads included raw sequencing data demultiplexing, amplicon sequence variants (ASVs) determination, data trimming, chimeric contigs removal, ASVs taxonomic classification using the SILVA database SSU release 138.
Data source location	Institution: Kuwait Institute for Scientific ResearchCity/Town/Region: KuwaitCountry: Kuwait • Latitude and longitude (28.63 N 47.93 E, 29.13 N 47.81 E, 28.79 N 47.58 E), Kuwait.
Data accessibility	Repository name: Mendeley Data Data identification number: DOI: 10.17632/wxfj336dv9.1 Direct URL to data: https://data.mendeley.com/datasets/wxfj336dv9/1 Repository name: National Centre for Biotechnology Information (NCBI) Data identification number: Accession: PRJNA814013, ID: 814013 Direct URL to data: https://www.ncbi.nlm.nih.gov/search/all/?term=PRJNA814013
Related research article	Rita Rahmeh^1^*, Abrar Akbar^1^, Husam Alomirah^1^, Mohamed Kishk^1^, Abdulaziz Al-Ateeqi^1^, Salah Al-Milhm^1^, Anisha Shajan^1^, Batool Akbar^1^, Shafeah Al-Merri^1^, Mohammad Alotaibi^1^, Alfonso Esposito^2^. Camel milk microbiota: a culture-independent assessment DOI: 10.1016/j.foodres.2022.111629

## Value of the Data

• This dataset provides a description of the effect of the geographical locations and season on camel milk's bacterial diversity based on high-throughput sequencing of 16S rRNA gene amplicons.
• The generated data will serve the ministry of health, farmers, and persuade investors to develop camel milk-based products.
• These data can be used to compare the milk bacterial diversity between different milk producing species and camels from different regions.
• This finding is important for the development of camel milk based dairy products with enhanced quality and safety.
• The data can be used to design an appropriate control program in the camel dairy herd and to improve camel rearing.

## 1 Objective

This work aimed to study the camel milk's bacterial diversity among samples groups from different geographical locations and seasons. This data article describes the sequences filtering statistics of all samples, the bacterial richness, the distribution of alpha diversity among sample groups and the differentially abundant bacteria at the phylum, class, order, family and genus level among the three locations and seasons.

## 2 Data Description

NGS raw data are available at https://www.ncbi.nlm.nih.gov/bioproject/PRJNA814013 and in Mendeley Data [2].

Recent studies reported the Bacterial diversity in goat, yak and cattle milk microbiome using high-throughput sequencing [3], [4], [5]. The aim of this dataset was to determine the impact of geographical locations and seasons on the bacterial population diversity in raw camel milk using high-throughput sequencing of the V3–V4 region of the 16S rRNA gene. The sequences filtering statistics of all samples are described in Mendeley Data [2]. A total of 15.68 million total read pairs was obtained and a total of 13.71 million high-quality 16S rRNA gene sequences (ASVs) were retained for the samples. The taxon frequencies of the most dominant bacterial genera (Taxa with a mean frequency at least 1%) summarized by class and genus between different geographical locations as well as seasons are available at Rahmeh et al. (2022) [1].

**Fig. 1:**
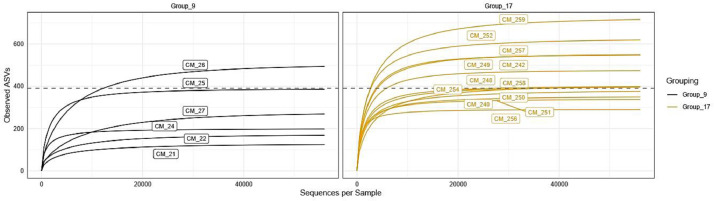
Alpha diversity analysis. Rarefaction curves among sample groups of raw camel milk collected during two seasons based on observed ASVs in individual samples. Group_9 was collected at season 1 (20°C) and Group_17 was collected at season 2 (37°C).

**Fig. 2:**
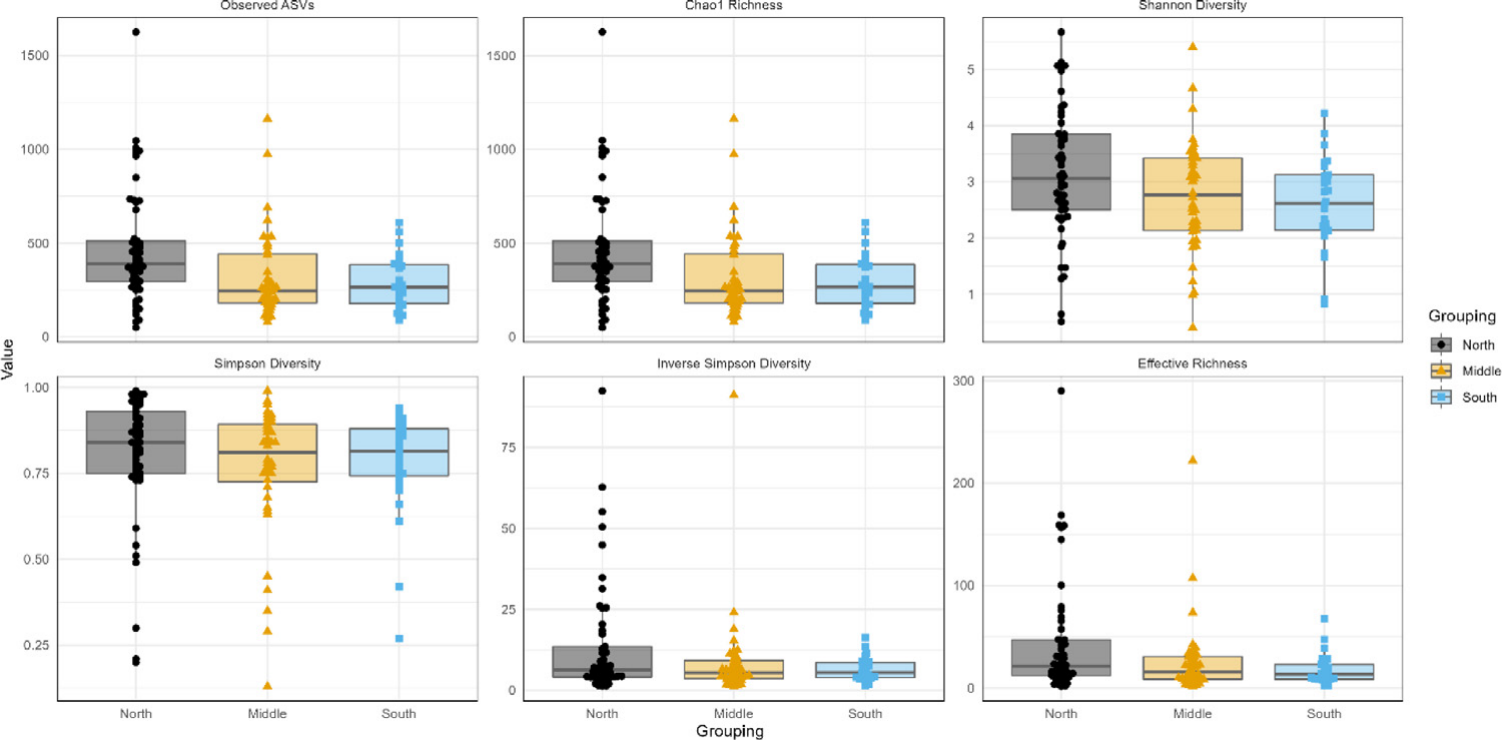
Alpha diversity analysis. Distribution of alpha diversity measures among camel milk sample groups collected from different geographical locations (north, middle, south Kuwait)

**Fig. 3:**
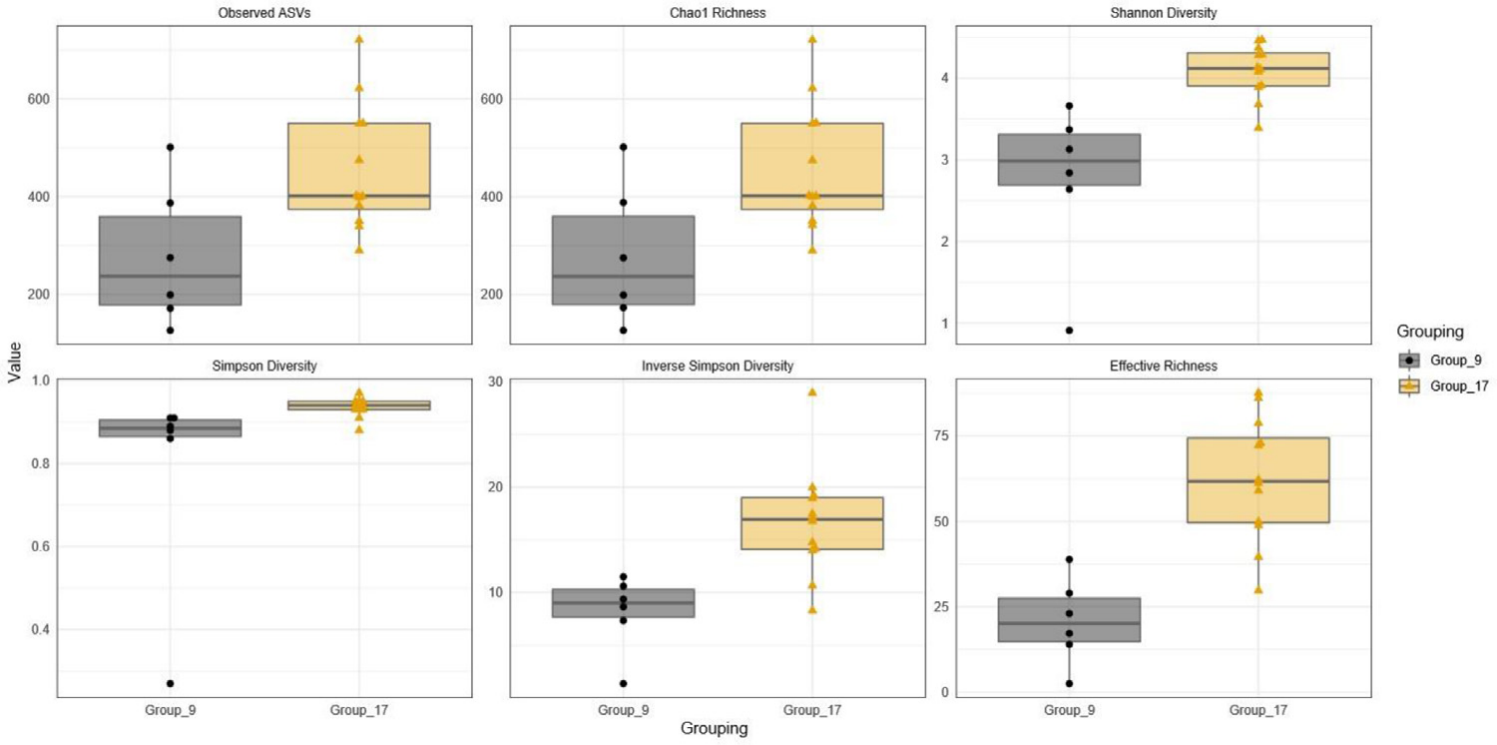
Alpha diversity analysis. Distribution of alpha diversity measures among camel milk sample groups collected during two seasons. Group_9 was collected at season 1 (20°C) and Group_17 was collected at season 2 (37°C).

Bacterial richness was evaluated by rarefaction curves among sample groups based on observed ASVs in individual samples. Rarefaction curves measure ASVs observed with a given depth of sequencing, and are used to compare observed richness among communities that have been unequally sampled [6]. The rarefaction curves for all samples were saturated and reached a plateau, suggesting that the sequencing depth was enough to capture the majority of the bacteria present in raw camel milk. Rarefaction curves among sample groups from two seasons are shown in Fig. 1. The distribution of alpha diversity measures among sample groups is presented in Figs. 2 and 3 for different geographical locations and seasons, respectively. Shannon and Simpson diversity indices were the highest for Group_17 (samples collected at season 2 (37°C)) and the samples collected from north Kuwait were more diverse than those from south and middle Kuwait.

**Fig. 4:**
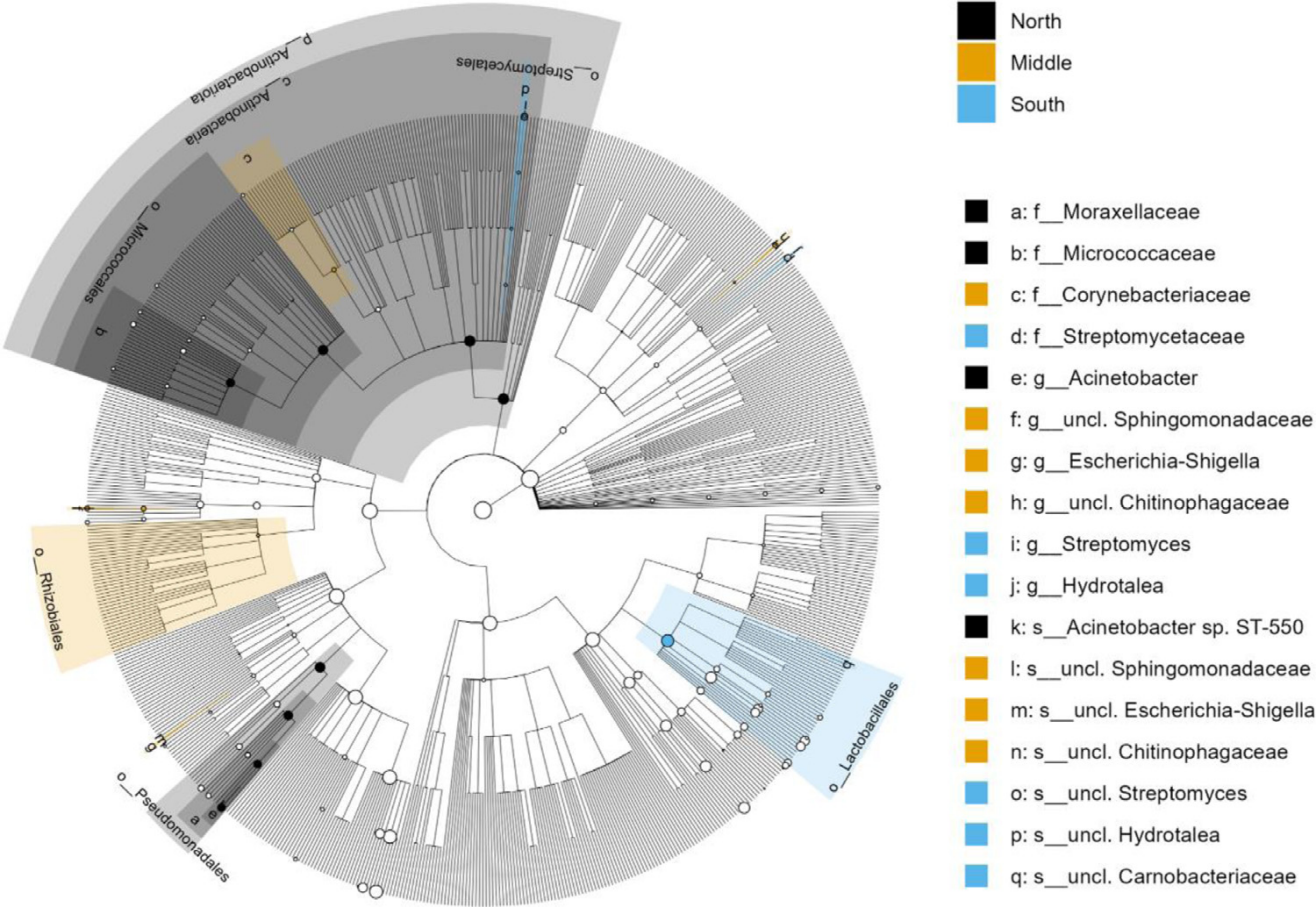
Cladogram of the top ten marker taxa. Cladogram of the top ten marker per sample group per geographical location (north, middle, south Kuwait)

**Fig. 5:**
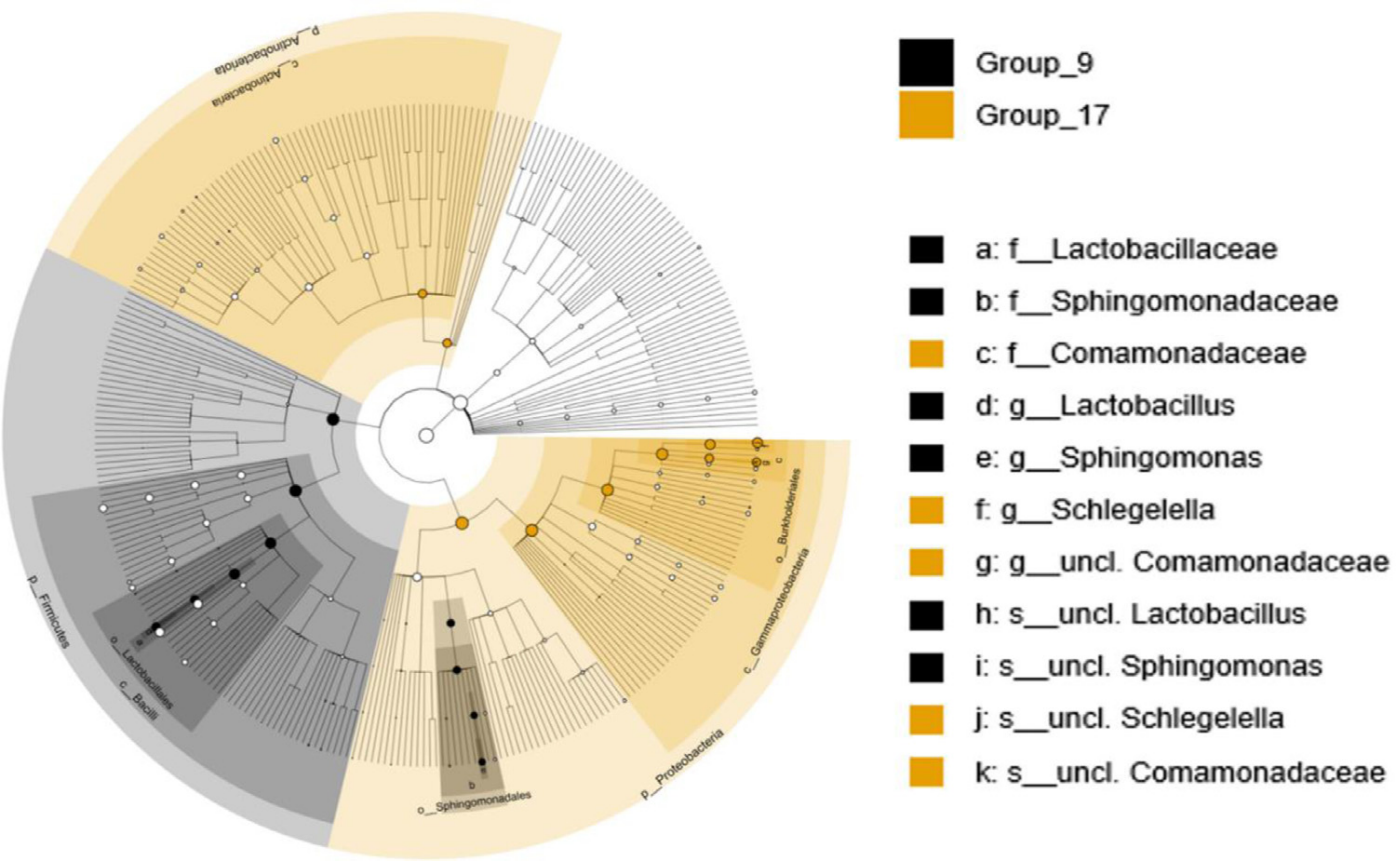
Cladogram of the top ten marker taxa. Cladogram of the top ten marker per sample group per season. Group_9 was collected at season 1 (20°C) and Group_17 was collected at season 2 (37°C).

LefSe was used to perform differentially abundant analysis at the phylum, class, order, family and genus level between geographical locations and seasons. LefSe showed a significant difference in the bacterial population in camel milk between the geographical locations, as well as the two seasons. Effect size and statistical significance per geographical location and season at the phylum, class, order, family and genus level are shown in Mendeley Data [2]. This analysis identified a total of 62, 16 and 5 differentially abundant genera in the north, middle and south, respectively, with P value < 0.05. In season 1, 6 differentially abundant genera were identified and 34 differentially abundant genera at season 2, with P value < 0.05. Cladograms of the top ten marker taxa per sample group per geographical location and season are visualized in Figs. 4 and 5, respectively. As shown in Fig. 4, the genus *Acinetobacter* was more enriched in the north and *Escherichia*-*Shigella* was more enriched in the middle. In the south, the genera *Hydrotalea* and *Streptomyces* were more enriched. As shown in Fig. 5, the genera *Lactobacillus* and *Sphingomonas* were more enriched in season 1. However, *Schlegelella* and *unclassified Comamonadaceae* were more enriched in season 2.

## 3 Experimental Design, Materials and Methods

### 3.1 Camel milk collection, DNA isolation and high throughput sequencing

A total of one hundred thirty-three samples were collected by manually milking one hundred thirty-three individual dromedary camels from south (forty samples), middle (forty samples) and north (fifty-three samples) Kuwait during two seasons (season 1, Autumn (20°C) and season 2, Summer (37°C)). The udder and teats were physically cleaned with 70% ethylic alcohol before sampling, and the first droplets of camel milk were eliminated. The samples were collected in sterile tubes, transported in a cool box to the laboratory for immediate DNA extraction. Samples were divided into groups based on their geographical locations (north, middle, south) and seasons. Group_9 was collected at season 1 (20°C) and Group_17 was collected at season 2 (37°C) from south Kuwait. After completely mixing 2 ml of camel milk samples, the samples were centrifuged at 10,000 × g for 10 min. The fat layer was removed and the pellet was used for DNA extraction. DNA was extracted using GenElute Bacterial Genomic DNA Kit (Sigma-Aldrich, USA) according to the manufacturer's instructions. The concentration of the extracted DNA was determined using a Qubit 3.0 Fluorometer (Invitrogen, USA). The quality of the extracted DNA was assessed by electrophoresis in 0.8% agarose gel. The 16S V3‒V4 library was prepared using custom fusion primers including the appropriate P5/P7 Illumina adapter sequence, an 8-nt index sequence, and the gene-specific primer sequence for bacteria 341F (ACTCCTACGGGAGGCAGCAG) and 806R (GGACTACHVGGGTWTCTAAT). Libraries were purified with Agencourt AMPure XP beads (Beckman Coulter, Germany) and validated with an Agilent Technologies 2100 bioanalyzer. Sequencing was performed on Illumina HiSeq 2500 in 2  ×  300 bp mode.

### 3.2 Bioinformatics and statistical analysis

Bioinformatics processing of the raw reads was performed. Raw sequencing data were demultiplexed. The DADA2 pipeline (R package dada2 v1.20.0) was used to identify amplicon sequence variants (ASVs) [7]. Primer sequences within an edit distance of 3 were eliminated from 5’ and 3’ ends of input read pairs with the BBTools package v38.45 [8]. Forward reads with ≤ 2 expected errors and reverse reads with ≤ 4 expected errors were retained. Error-corrected reads with a minimum overlap of 20 bp were patched to contiguous sequences (contigs). The 'consensus' approach in DADA2 was used to delete chimeric contigs made up of two partial sequences of different origin. The IDTAXA approach [9] implemented in the R package DECIPHER v2.18.1 [10] was used to taxonomically classify the remaining contigs (ASVs) using the SILVA database SSU release 138 [11,12]. ASVs with a classification confidence value ≥ 51% were retained. Descriptive Alpha diversity was measured by the indices calculated with R package vegan v2.6.0 [13] as follows: Observed ASVs; Chao1 richness; Shannon diversity; Simpson diversity; Inverse Simpson diversity; and Effective richness 1D. Rarefaction curves were produced based on observed ASVs in individual samples. Linear discriminant analysis Effect Size (LEfSe) [14] was applied to ASV counts aggregated at different taxonomic ranks using R package microbiome Marker v0.0.1.9000 [15]. ASVs with at least ten counts in two or more samples were considered.

## Ethics Statements

All experimental protocols were approved by the Center Proposal Evaluation Committee (PEC) of Kuwait Institute for scientific research. All methods were performed in accordance with relevant institutional guideline and regulations with Reference No. PMO/PV/GM/073/2015, in compliance with the standards of animal rights and with camel owner's permission.

## CRediT Author Statement

**Rita Rahmeh:** Experimental design, Data analysis and results interpretation, Paper writing; **Husam Alomirah:** Experimental design, Data analysis; **Abrar Akbar:** DNA extraction and data analysis; **Mohamed Kishk, Abdulaziz Al-Ateeqi** and **Salah Al-Milhm:** Camel milk collection; **Mohammad Alotaibi, Anisha Shajan** and **Batool Akbar:** DNA extraction and data tabulation; **Shafeah Al-Merri:** Design of an application for camel data collection; **Alfonso Esposito:** Bioinformatics analysis. All co-authors co-wrote the paper.

## Declaration of Competing Interest

The authors declare that they have no known competing financial interests or personal relationships that could have appeared to influence the work reported in this paper.

## Data Availability

Camel milk Microbiome analysis (Original data) (Mendeley Data). Microbiome of Raw Camel Milk (Reference data) (National Center for Biotechnology Information).
